# Improving maize grain yield by matching maize growth and solar radiation

**DOI:** 10.1038/s41598-019-40081-z

**Published:** 2019-03-06

**Authors:** Yunshan Yang, Wenjuan Xu, Peng Hou, Guangzhou Liu, Wanmao Liu, Yonghong Wang, Rulang Zhao, Bo Ming, Ruizhi Xie, Keru Wang, Shaokun Li

**Affiliations:** 1 Key Laboratory of Oasis Ecology Agriculture of Xinjiang Construction Corps, The Center of Crop High-Yield Research, Shihezi University, Shihezi, 832003 China; 2 Key Laboratory of Crop Physiology and Ecology, Institute of Crop Sciences, Chinese Academy of Agricultural Sciences, Ministry of Agriculture, Beijing, 100081 China; 3 Key Laboratory of Southwest China Wildlife Resources Conservation, Ministry of Education, College of life Sciences, China West Normal University, Nanchong, 637002 Sichuan China; 4 Institute of Crop Sciences, Ningxia Academy of Agricultural and Forestry Sciences, Yinchuan, 750105 Ningxia Hui Autonomous Region China

## Abstract

Matching of maize growth with solar radiation is of great importance for achieving high yield. We conducted experiments using different maize cultivars and planting densities under different solar radiations during grain filling to quantitatively analyze the relationships among these factors. We found that a decrease in solar radiation after silking caused a drop in maize grain yield and biomass, with lower solar radiation intensities leading to worse grain yields and biomass. Cultivar ZD958 was more sensitive to solar radiation changes than cultivar XY335; slight decreases in solar radiation (i.e., 15% shading) caused significant declines in ZD958 grain yield. When total solar radiation during grain filling was less than 486.9 MJ m^−2^ for XY335 and less than 510.9 MJ m^−2^ for ZD958, the two cultivars demonstrated high yields at lower planting density of 7.5 × 10^4^ plants ha^−1^; average yields were 13.36 and 11.09 Mg ha^−1^, respectively. When radiation intensities were higher than 549.5 MJ m^−2^ for XY335 and higher than 605.8 MJ m^−2^ for ZD958, yields were higher at a higher planting density of 12 × 10^4^ plants ha^−1^, with average yields of 20.58 Mg ha^−1^ for XY335 and 19.65 Mg ha^−1^ for ZD958.

## Introduction

Food security is fundamental for human survival and national security. By 2050, the global population is expected to increase to 9.2 billion from 6.5 billion today^1^. Population growth and expanding consumption will drive global food demand up by 50% by 2030, or double that of 2000 levels^2^. A primary strategy for maintaining food security in China and the rest of the world is to ensure adequate food supplies. Studies have shown that China’s future arable land is expected to decrease, whereas food consumption and demand are expected to continue increasing^3^. In the absence of major breakthroughs in farming systems and agricultural production, the most viable strategy to ensure food security is to continuously increase crop yields^4^. However, maize yield in China is lower than that in the Europe and the U.S.^5^. Adapting maize growth to the environment will be an important innovation which will match maize growth and the climate (solar radiation, temperature etc.) and increase total biomass and then grain yield.

Solar radiation drives crop photosynthesis, the formation and development of plant organs, and crop yields. Solar radiation is a primary determinant of potential crop productivity^6–9^, so increases in crop yields can be achieved by improving radiation use efficiency. Maize is a high light-use efficiency crop, with more than 95% of its dry matter formed through photosynthesis^10^. Modern maize production is based on high planting densities^11^. Climatic conditions, especially solar radiation, significantly affect maize growth and optimal planting densities^12–14^. Maize cultivation is widespread in the world and solar radiation and planting densities vary significantly among different maize planting countries and regions^14–16^. For example, in China solar radiation in the west region is more abundant than that in eastern region. As a result, grain yields and planting densities in the west are higher than in the east^14,17^. In recent years, due to climate warming, global solar radiation has shown a decreasing trend, with average declines of 1.4–2.7% per decade^17–20^. China is one country that has experienced severe declines in solar radiation, with total solar radiation decreasing by 4.5 W m^−2^ per decade and effective sunshine hours decreasing by 1.28% per decade on average^17^. These declines are most severe in eastern China where total solar radiation has decreased by more than 6% per decade^18–20^. Declining solar radiation significantly affects maize growth at all growing stages, especially during grain filling and thus decreases grain yield^21,22^.

Most researches have shown the effects of solar radiation on maize growth by conducting shading experiments^13,21–23^. Furthermore, different degrees and periods of shading have different effects on maize growth and development^23^. Previous studies had shown that shading caused maize grain yields to decrease significantly. In general, reductions in maize grain yields are greater with post-silking shading than before-silking shading^21,24,25^. Moreover, different maize genotypes show different responses to shading in terms of their growth and development. For maize varieties that are sensitive to weak light, the photosynthetic rate and fluorescence parameters of their leaves decrease greatly with shading, whereas varieties that are insensitive to weak light show only slight decreases in these two parameters. However, most previous studies were conducted in low solar radiation areas^13^, so any shading experiments performed in these areas would result in radiation levels below those that exist in any maize planting regions in China and other countries and therefore have little significance in real situations^21–23^. Furthermore, few studies have investigated the interactive and quantitative relationships between solar radiation, planting density, and genotype. Therefore, Qitai and Yinchuan where the solar radiation resources were the most abundant in China were chosen as the experimental sites in this study which would be more meaningful for studying the effects of different solar radiation on maize growth by conducting different shading level experiments. Through experiments of different solar radiation levels during grain filling, planting densities, and genotypes, we studied the interactions and quantitative relationships between these factors and explored optimal planting densities and genotypes under different solar radiation conditions. Our hypotheses are: (1) as solar radiation increases, optimal planting density also increases and (2) different genotypes respond differently to changes in solar radiation. This study can inform best practices for achieving high-yield maize cultivation in regions with different solar radiation intensities in China and other countries in the world as well as in the face of climate change.

## Results

### Effects of different shading levels on maize grain yield

**Figure 1:**
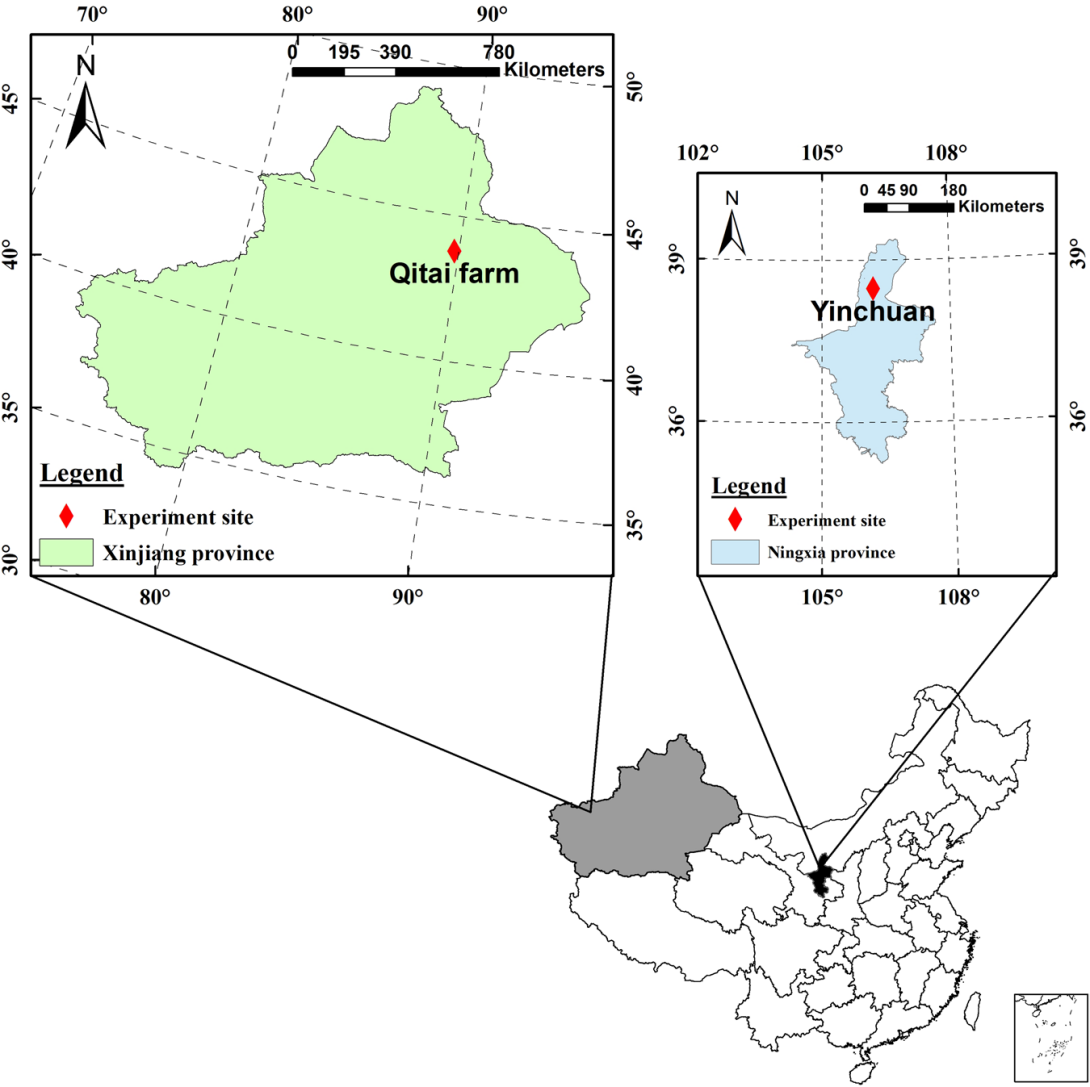
The positions of two experimental sites.

**Figure 2:**
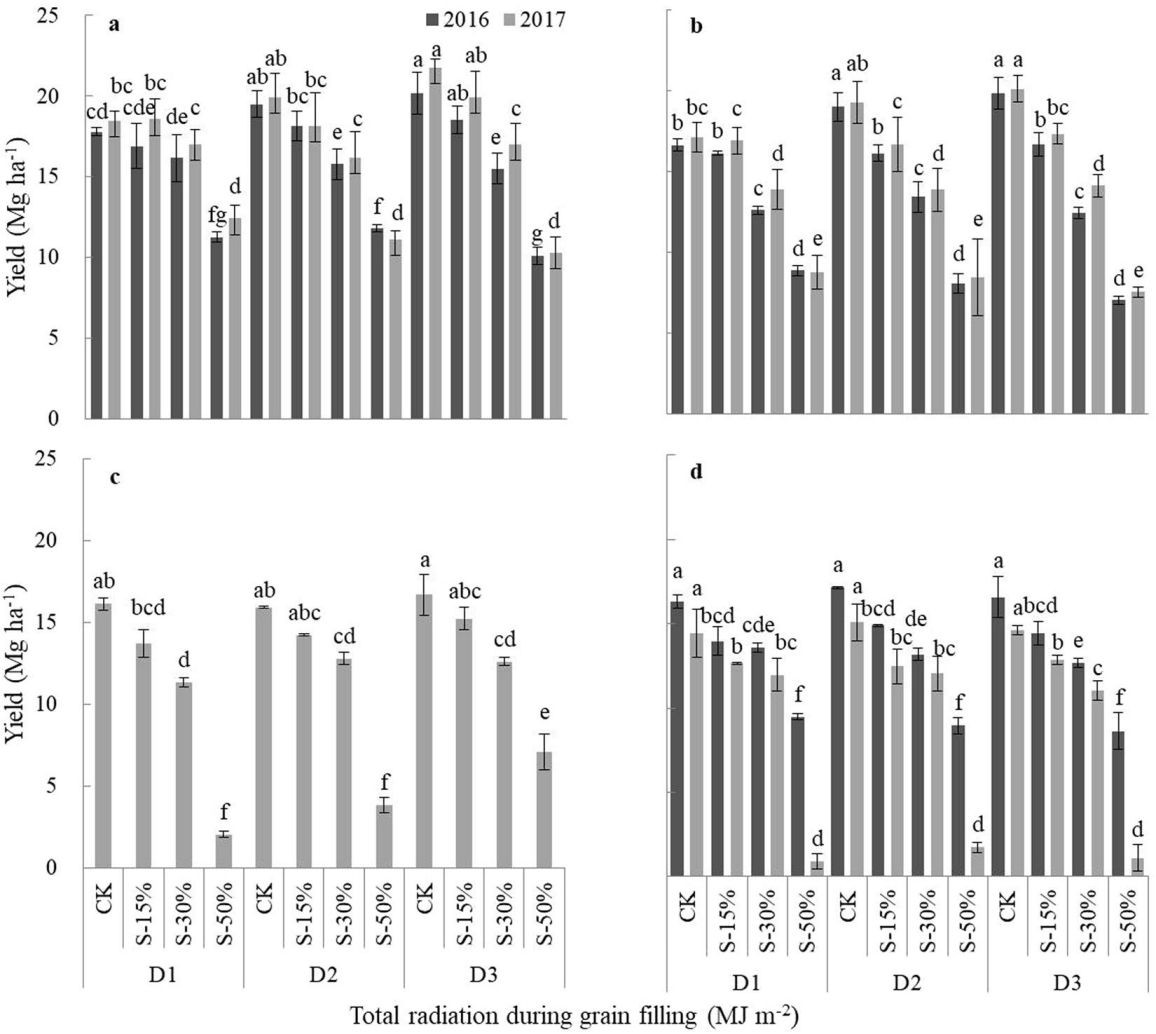
Effects of different shading levels (CK, S-15%, S-30%, S-50%) and densities (D1, D2, D3) on maize grain yields. (**a**) (Qitai, XY335), (**b**) (Qitai, ZD958), (**c**) (Yinchuan, XY335), (**d**) (Yinchuan, ZD958). Note: Different lowercase letters indicate significant differences between treatments at *P* < 0.05.

The results of the current study suggest that shading leads to accelerated maize senescence. The duration of maize crop growth was longer at Qitai than at Yinchuan (Table 1) and was longer for ZD958 than for XY335, and the positions of two experimental sites were shown in Fig. 1. As shown in Table 2, the average temperature at Yinchuan was higher than at Qitai, while solar radiation was lower. Table 3 presents the specific experimental parameters. Experimental results at the two sites were averaged for 2016 and 2017. Under natural light condition (CK), XY335 grain yield was higher than that of ZD958 (Fig. 2). The grain yields of both maize cultivars decreased as shading increased, but shading affected maize grain yields differently depending on planting density. The S-15% shading treatment produced no significant difference in XY335 grain yield compared with CK under all planting density treatments. We also found no significant difference in XY335 grain yield between the S-30% and CK treatments at the D1 planting density. However, XY335 yield at S-50% and D1 was 34.7% less than under the CK condition. At the D2 planting density, XY335 yields at S-30% and S-50% were 19.2% and 41.8% lower than yields under the CK condition, respectively. At the D3 planting density, XY335 yields were 23.6% and 51.3% lower than CK yields at S-30% and S-50%, respectively. In contrast, ZD958 yields under all shading treatments were significantly lower than for CK at all planting densities. ZD958 yields under S-15%, S-30%, and S-50% were 13.3%, 19.3%, and 50.1% lower, respectively, than the CK condition at the D1 planting density. These values were 14.8%, 25.2%, and 54.7% lower than the CK condition under the D2 planting density and 13.7%, 28.7%, and 63.5% lower under the D3 planting density. Our findings that XY335 displayed no significant yield differences between CK, S-15%, and S-30% at low planting densities, but ZD958 displayed significantly lower yields at all shading treatments at all planting densities indicate that ZD958 is more sensitive to reductions in solar radiation.

**Table 1:** Phenological development of XY335 and ZD958 at different shading levels (CK = natural light; S-15% = 85% of natural light; S-30% = 70% of natural light; S-50% = 50% of natural light) and densities (D1 = 7.5 × 10^4^ plants ha^−1^; D2 = 10.5 × 10^4^ plants ha^−1^; and D3 = 12 × 10^4^ plants ha^−1^) at Qitai and Yinchuan in 2016 and 2017.

Site	Cultivar	Planting density	Shading level	Qitai				Yinchuan			
Year				Sowing Date	Silking Date	Physiological maturity Date	Growth duration (d)	Sowing Date	Silking Date	Physiological maturity Date	Growth duration (d)
2016	XY335	D1	CK	4/18	7/11	9/23	158	—	—	—	—
			S-15%	4/18	7/11	9/23	158	—	—	—	—
			S-30%	4/18	7/11	9/23	158	—	—	—	—
			S-50%	4/18	7/11	9/20	155	—	—	—	—
		D2	CK	4/18	7/12	9/25	160	—	—	—	—
			S-15%	4/18	7/12	9/25	160	—	—	—	—
			S-30%	4/18	7/12	9/23	158	—	—	—	—
			S-50%	4/18	7/12	9/20	155	—	—	—	—
		D3	CK	4/18	7/12	9/25	160	—	—	—	—
			S-15%	4/18	7/12	9/25	160	—	—	—	—
			S-30%	4/18	7/12	9/23	158	—	—	—	—
			S-50%	4/18	7/12	9/19	154	—	—	—	—
	ZD958	D1	CK	4/18	7/14	9/28	163	4/21	7/4	9/24	156
			S-15%	4/18	7/14	9/28	163	4/21	7/4	9/23	155
			S-30%	4/18	7/14	9/26	161	4/21	7/4	9/23	155
			S-50%	4/18	7/14	9/23	158	4/21	7/4	9/20	152
		D2	CK	4/18	7/16	9/28	163	4/21	7/5	9/24	156
			S-15%	4/18	7/16	9/27	162	4/21	7/5	9/23	155
			S-30%	4/18	7/16	9/24	159	4/21	7/5	9/23	155
			S-50%	4/18	7/16	9/21	156	4/21	7/5	9/20	152
		D3	CK	4/18	7/16	9/28	163	4/21	7/5	9/24	156
			S-15%	4/18	7/16	9/25	160	4/21	7/5	9/22	154
			S-30%	4/18	7/16	9/23	158	4/21	7/5	9/18	150
			S-50%	4/18	7/16	9/19	154	4/21	7/5	9/16	148
2017	XY335	D1	CK	4.21	7/9	9/26	158	4/20	7/4	9/20	152
			S-15%	4.21	7/9	9/26	158	4/20	7/4	9/20	152
			S-30%	4.21	7/9	9/26	158	4/20	7/4	9/18	150
			S-50%	4.21	7/9	9/23	155	4/20	7/4	9/14	146
		D2	CK	4.21	7/10	9/28	160	4/20	7/6	9/20	152
			S-15%	4.21	7/10	9/28	160	4/20	7/6	9/20	152
			S-30%	4.21	7/10	9/24	156	4/20	7/6	9/16	148
			S-50%	4.21	7/10	9/21	153	4/20	7/6	9/14	146
		D3	CK	4.21	7/11	9/28	160	4/20	7/7	9/23	155
			S-15%	4.21	7/11	9/28	160	4/20	7/7	9/20	152
			S-30%	4.21	7/11	9/25	157	4/20	7/7	9/18	150
			S-50%	4.21	7/11	9/20	152	4/20	7/7	9/16	148
	ZD958	D1	CK	4.21	7/9	9/29	161	4/20	7/4	9/24	156
			S-15%	4.21	7/9	9/29	161	4/20	7/4	9/22	154
			S-30%	4.21	7/9	9/25	157	4/20	7/4	9/23	155
			S-50%	4.21	7/9	9/22	154	4/20	7/4	9/18	150
		D2	CK	4.21	7/10	9/29	161	4/20	7/4	9/24	156
			S-15%	4.21	7/10	9/30	162	4/20	7/4	9/22	154
			S-30%	4.21	7/10	9/25	157	4/20	7/4	9/22	154
			S-50%	4.21	7/10	9/21	153	4/20	7/4	9/18	150
		D3	CK	4.21	7/13	9/30	162	4/20	7/6	9/25	157
			S-15%	4.21	7/13	9/30	162	4/20	7/6	9/23	155
			S-30%	4.21	7/13	9/25	157	4/20	7/6	9/20	152
			S-50%	4.21	7/13	9/21	153	4/20	7/6	9/18	150

“—” Indicates that XY335 was not planted at Yinchuan in 2016.

**Table 2:** North latitude, east longitude, altitude, mean daily temperature, diurnal temperature variation, solar radiation, and precipitation during the maize growing season at Qitai and Yinchuan in 2016 and 2017.

Years	Site	North latitude	East longitude	Altitude (m)	Mean daily temperature (°C)	Diurnal temperature variation (°C)	precipitation (mm)	Accumulated solar radiation (MJ m^−2^)
2016	Qitai	43°50′	89°46′	1020	20.08	14.12	176.3	1571
2017					20.35	15.54	107.8	1592
2016	Yinchuan	38°13′	106°14′	1120	21.53	12.12	198.6	1476
2017					21.78	12.91	142.5	1503

**Table 3:** Experimental treatments for different shading levels (CK, S-15%, S-30%, S-50%) and densities (D1, D2, D3) in 2016 and 2017.

Years	Site	Cultivar		Planting density			Shading level			
2016	Qitai	XY335	ZD958	D1	D2	D3	CK	S-15%	S-30%	S-50%
	Yinchuan	—	ZD958							
2017	Qitai	XY335	ZD958							
	Yinchuan	XY335	ZD958							

“—” Indicates that XY335 was not planted at Yinchuan in 2016.

In 2016, XY335 grain yields in plots planted at the D1 density and under the CK, S-15%, and S-30% conditions were significantly higher than the grain yield under S-50% at Qitai; however, when planted at the D2 and D3 densities, XY335 yields under the CK and S-15% conditions were significantly higher than under S-30% and S-50%. For ZD958, yields under the CK and S-15% conditions were significantly higher than under S-30% and S-50% at the D1 planting density; however, yields under the different shading treatments at the D2 and D3 planting densities decreased in the order CK > S-15% > S-30% > S-50%. Shading produced similar changes in yields for the two cultivars, although ZD958 yield reductions were more significant. At the same planting densities, ZD958 grain yields at Yinchuan under different shading treatments decreased in the order: CK > S-15% > S-30% > S-50%.

In 2017, grain yields for the two maize cultivars at both sites in 2017 showed similar trends. Yields at Qitai in 2017 were slightly higher than in 2016 for the same treatments, whereas the opposite trend was observed at Yinchuan.

### Effects of different shading levels on maize biomass and leaf area index

The biomass of both maize cultivars decreased as shading increased (Table 4). Dry matter accumulations at the same planting densities and cultivar followed the order of S-CK > S-15% > S-30% > S-50%. As planting density increased, biomass increased for all shading treatments except S-50%. There was a significant difference in dry matter production for XY335 between the CK and S-50% treatments at the D1 planting density; at D2 and D3, dry matter production under the CK and S-15% treatments was significantly higher than under S-30% and S-50%. For ZD958, dry matter production at the D1 planting density under CK and S-15% was significantly higher than under the S-30% and S-50% treatments, while dry matter production at D2 and D3 under the different shading treatments decreased significantly in the order CK > S-15% > S-30% > S-50%. Dry matter production for the two cultivars showed a similar response to increased shading, although the dry matter reductions for ZD958 were more significant. The trend in dry matter production was similar to that of yield, which indicates that reductions in dry matter production with increased shading was the primary cause of reduced grain yields.

**Table 4:** Effects of different shading levels (CK, S-15%, S-30%, S-50%) and densities (D1, D2, D3) on biomass (at maturity) and LAI (leaf area index) after 40 days of shading at Qitai and Yinchuan in 2016 and 2017.

Cultivar	Planting density	Shading level	Biomass (t ha^−1^)				LAI			
			Qitai		Yinchuan		Qitai		Yinchuan	
			2016	2017	2016	2017	2016	2017	2016	2017
XY335	D1	CK	30.95 cd	37.59 ab	—	31.20 ab	5.07 fg	6.06 de	—	5.82 bc
		S-15%	31.21 cd	33.71 b	—	30.22 ab	4.82 fg	5.94 de	—	6.19 bc
		S-30%	29.69 d	34.17 b	—	29.97 b	4.80 fg	6.04 de	—	5.88 bc
		S-50%	25.87 e	17.95 c	—	24.38 c	4.63 g	5.56 e	—	6.01 bc
	D2	CK	37.27 ab	42.12 a	—	31.24 ab	7.25 ab	7.69 ab	—	6.59 ab
		S-15%	35.01 b	34.65 ab	—	29.84 b	6.78 ab	7.83 ab	—	6.74 ab
		S-30%	32.31 c	33.33 b	—	25.57 c	6.33 bc	7.98 a	—	6.05 bc
		S-50%	25.42 e	18.84 c	—	23.57 d	5.82 def	6.64 cd	—	5.19 cd
	D3	CK	39.72 a	40.15 a	—	32.13 a	7.77 a	8.38 a	—	7.41 ab
		S-15%	37.75 a	39.97 ab	—	30.39 ab	7.18 ab	8.21 a	—	6.84 ab
		S-30%	35.10 b	34.18 b	—	26.00 c	7.07 ab	8.07 a	—	5.21 cd
		S-50%	26.34 e	18.17 c	—	20.30 e	6.01 cd	7.04 bc	—	4.37 d
ZD958	D1	CK	30.57 de	32.20 bc	30.89 ab	30.83 ab	5.06 d	5.55 e	5.78 bc	4.68abc
		S-15%	29.00 e	30.72 bcd	27.79 bcd	30.27 b	5.41 d	5.39 e	5.71 bc	3.83 abc
		S-30%	25.56 f	24.61 e	22.38de	29.15 c	5.49 d	5.30 e	5.49 bc	3.79 cde
		S-50%	19.99 h	14.30 f	19.59 e	22.46 e	5.39 d	5.10 e	4.97 c	3.25 de
	D2	CK	35.69 b	33.17 bc	32.37 a	31.10 a	7.37 abc	7.57 ab	6.22 b	4.99 ab
		S-15%	32.74 c	31.23 bcd	23.08 d	29.16 c	7.28 abc	7.31 bc	6.26 b	4.67 abc
		S-30%	30.83 d	28.94 d	21.98 de	22.47 d	7.10 abc	7.06 bcd	6.12 b	4.28 abc
		S-50%	23.75 g	15.88 f	20.81 e	22.09 e	5.79 cd	6.66 d	5.06 c	3.35 de
	D3	CK	37.47 a	39.88 a	31.23 ab	31.81 a	8.69 a	7.51 ab	7.38 a	5.38 a
		S-15%	34.18 bc	34.85 b	27.47 c	26.18 cd	7.50 ab	7.84 a	6.56 b	5.29 a
		S-30%	30.28 de	30.04 cd	22.07 de	22.48 d	7.95 ab	7.23 bc	5.17 c	4.08 bc
		S-50%	23.11 g	23.23 e	20.59 e	20.47 f	6.69 bcd	6.79 cd	4.62 c	2.63 e

“—” Indicates that XY335 was not planted at Yinchuan in 2016. Note: The lowercase letters in the figure indicate significant differences at a 5% level.

The LAI of the two cultivars showed similar drecreasing trends with increased shading, although the trend was more significant for ZD958 (Table 4). There were no significant differences in the LAIs of the two cultivars at the different shade treatments at the D1 planting density. When planted at the D2 density, the LAIs of the two cultivars at S-50% were significantly lower than those of the other treatments. At the D3 density, the LAIs of the two cultivars at S-30% and S-50% were significantly lower than those at CK and S-15%. This indicates that leaves are more sensitive to changes in solar radiation at high planting densities and that low solar radiation accelerates leaf senescence at high planting densities.

### Genotype, planting density, solar radiation, and their interactive effects on maize yield

**Figure 3:**
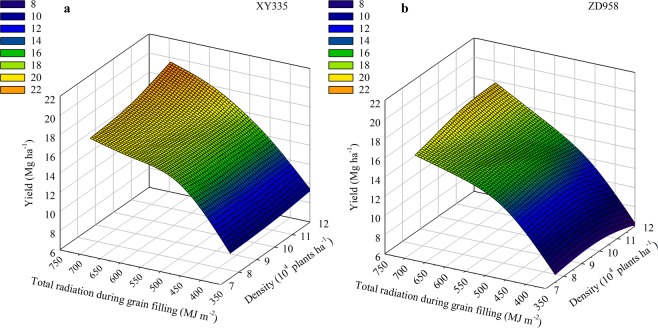
The interactive effects of genotype, planting density, and solar radiation on maize yield.

Figure 3 shows the interactive effects of planting density and solar radiation on maize grain yields. Grain yields of the two cultivars increased as solar radiation increased during grain filling. Under the same amount of solar radiation, XY335 yields were higher than ZD958 yields. Furthermore, ZD958 required more solar radiation to produce the same grain yield as XY335. This indicates that the solar radiation use efficiency of XY335 was much higher than that of ZD958. When solar radiation levels during grain filling were in the ranges of 350–370, 370–410, 410–460, 460–555, and 555–615 MJ m^−2^, the corresponding XY335 yields were about 10, 12, 14, 16, and 18 Mg ha^−1^, respectively. When the solar radiation during grain filling was higher than 615 MJ m^−2^ and the planting density was 7.5 × 10^4^–9.7 × 10^4^ plants ha^−1^, grain yield was about 8 Mg ha^−1^; when the planting density gradually increased to 12 × 10^4^ plants ha^−1^, grain yield increased to about 22 Mg ha^−1^. For ZD958, when solar radiation during grain filling was in the ranges of 350–370, 370–415, 415–460, 460–530, and 530–630 MJ m^−2^, the corresponding yields were about 8, 10, 12, 14, and 16 Mg ha^−1^, respectively. When the solar radiation during grain filling was higher than 630 MJ m^−2^ and the planting density was 12 × 10^4^ plants ha^−1^, grain yield was about 20 Mg ha^−1^.

We conclude that when the solar radiation during grain filling is less than 623 MJ m^−2^, increases in maize yield are primarily related to the amount of solar radiation during grain filling. In other words, the greater the solar radiation during grain filling, the greater the grain yield. When the solar radiation during grain filling is higher than 623 MJ m^−2^, increases in maize yield are primarily related to planting density. Therefore, low planting densities are more suitable for areas where the solar radiation during grain filling is less than 623 MJ m^−2^. However, in areas receiving greater than 623 MJ m^−2^ solar radiation during grain filling, radiation use efficiency can be improved by increasing planting density. XY335 yields gradually increased with increasing planting density, and did so more significantly than ZD958 yields. When the solar radiation during grain filling was higher than 623 MJ m^−2^, XY335 grain yield was higher than that of ZD958 at the same planting density. The maximum grain yield for ZD958 occurred at the planting density of 9.5 × 10^4^–10.5 × 10^4^ plants ha^−1^. However, when the planting density increased, yield decreased. This indicates that XY335 has a higher yield potential than ZD958 under sufficient solar radiation conditions. When solar radiation is not a constraint, XY335 grain yield could be improved by increasing planting density.

### Quantitative relationship between maize yield and solar radiation

**Figure 4:**
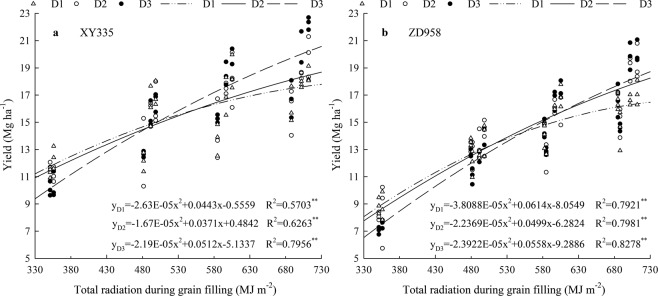
Quantitative relationship between maize yield and solar radiation under different planting densities (D1, D2, D3). Notes: R, Pearson’s correlation coefficient, **Significant at the level of *P* < 0.01.

Using the regression equation, we concluded that the solar radiation levels during grain filling corresponding with the highest XY335 yields at the D1, D2, and D3 planting densities were: 18.09 Mg ha^−1^ and 841.9 MJ m^−2^, 20.13 Mg ha^−1^ and 1110.2 MJ m^−2^, and 24.79 Mg ha^−1^ and 1168.9 MJ m^−2^, respectively. Under the same conditions the corresponding values for ZD958 were: 16.69 Mg ha^−1^ and 806.02 MJ m^−2^, 21.55 Mg ha^−1^ and 1115.38 MJ m^−2^, and 23.25 Mg ha^−1^ and 1166.29 MJ m^−2^. Under the same planting densities XY335 yields were generally higher than those of ZD958. In addition, we found that yield increases with increasing planting density until a maximum yield is reached. This maximum yield and corresponding density are determined by the amount of radiation. The higher the radiation, the higher the density and yield. In addition, XY335 required less solar radiation to produce the same grain yield as ZD959, which indicates that the radiation use efficiency of XY335 was higher than that of ZD958 (Fig. 4). For XY335, when the solar radiation during grain filling was less than 486.9 MJ m^−2^, yields decreased in the order D1 > D2 > D3 (average yields were 13.36 Mg ha^−1^, 13.03 Mg ha^−1^, and 12.4 Mg ha^−1^, respectively); when the solar radiation during grain filling was 486.9–502.4 MJ m^−2^, yields decreased in the order D1 > D3 > D2 (average 17.45 Mg ha^−1^, 17.36 Mg ha^−1^, and 14.54 Mg ha^−1^); when the solar radiation during grain filling was 502.4–549.5 MJ m^−2^, yields decreased in the order D3 > D1 > D2 (average 15.72 Mg ha^−1^, 15.45 Mg ha^−1^, and 15.37 Mg ha^−1^); when the solar radiation during grain filling was higher than 549.5 MJ m^−2^, yields decreased in the order D3 > D2 > D1 (average 20.58 Mg ha^−1^, 17.98 Mg ha^−1^, and 16.97 Mg ha^−1^). For ZD958, when the solar radiation during grain filling was less than 510.9 MJ m^−2^, yields decreased in the order D1 > D2 > D3 (average 11.09 Mg ha^−1^, 10.82 Mg ha^−1^, and 10.14 Mg ha^−1^); when the solar radiation during grain filling was in the range of 510.9–552.6 MJ m^−2^, yields decreased in the order D2 > D1 > D3 (average 13.91 Mg ha^−1^, 13.80 Mg ha^−1^, and 13.59 Mg ha^−1^); when the solar radiation during grain filling was 552.6–605.8 MJ m^−2^, yields decreased in the order D2 > D3 > D1 (average 16.89 Mg ha^−1^, 16.77 Mg ha^−1^, and 14.71 Mg ha^−1^); when the solar radiation during grain filling was higher than 605.8 MJ m^−2^, yields decreased in the order D3 > D2 > D1 (average 19.65 Mg ha^−1^, 19.23 Mg ha^−1^, and 16.03 Mg ha^−1^). This demonstrates that under different solar radiation conditions, appropriate planting densities are a prerequisite for high yields. In summary, for XY335 and ZD958, when the solar radiation during grain filling was less than 486.9 MJ m^−2^ and 510.9 MJ m^−2^, respectively, the appropriate planting density was 7.5 × 10^4^ plants ha^−1^; when the soalr radiation during grain filling was 486.9–502.4 MJ m^−2^ and 510.9–552.6 MJ m^−2^, respectively, the appropriate planting density was 10.5 × 10^4^ plants ha^−1^; and when the solar radiation during grain filling was higher than 549.5 MJ m^−2^ and 605.8 MJ m^−2^, respectively, the appropriate planting density was 12 × 10^4^ plants ha^−1^. Comparing the two cultivars, we found that under the same planting densities and solar radiation levels, ZD958 yields were lower than those of XY335 and the yield differences between different planting densities were greater for ZD958 than for XY335.

## Discussion

Most studies on the effects of solar radiation on maize growth have examined shading treatments^15,22–25^. These studies have shown that reductions in solar radiation resulted in reduced maize yields and that the maize yield reductions increased gradually with decreasing solar radiation^26^. The largest yield reductions in summer maize yields due to shading occurred during silking^27–29^. In this study, maize yield decreased with decreasing solar radiation. Under the S-50% treatment, grain yield was the lowest. In addition, high density planting is an effective way to increase maize yields in modern maize production, but excessively high planting densities can exacerbate inter-plant competition for limited resources and eventually reduce maize yields. This study shows that reductions in solar radiation at different planting densities leads to different degrees of reduced yield, and that yield reduction increased with increasing planting density. For example, grain yield at the D3 planting density was the lowest under the lowest solar radiation condition of S-50% treatment. This is mainly because of that after shading and increasing planting densities, solar radiation in the canopy decreased sharply and inter-plant competition for limited solar radiation resources was exacerbated, and photosynthetic capacity of leaves decreased especially the lower leaves in the canopy^14–16^.

While rice and wheat yields have improved largely because of an increasing harvest index since the Green Revolution, maize yield increases are mainly attributed to large increases in total aboveground dry matter accumulation^2,4,9^. Previous studies have shown that maximum total biomass decreases under shade and that yield and dry matter accumulation decreases to different degrees correspondingly^30^. This study demonstrates that shading caused dry matter production of the two cultivars to decrease, but the trend was more significant for ZD958. The decreasing trend in dry matter production was similar to that of grain yield, which indicates that dry matter production is a primary cause of changes in grain yield. LAI is an agronomic variable that summarizes the complex combination of internal and external factors that influence the structures of plant canopies, which are strongly influenced by interactions between plant genotype and environmental factors^31^. Previous studies have shown that maintaining a green canopy and delaying leaf senescence prolongs photosynthesis for grain filling, and that high planting densities can accelerate leaf senescence^32^. LAI and chlorophyll content have been shown to decrease after shading^28^. This study shows that LAI decreased under low light conditions. This indicates that leaves are more sensitive to changes in solar radiation especially at high planting densities and that low solar radiation at high planting densities can accelerate leaf senescence. LAI reduction of ZD958 was more significant than for XY335, which may have caused the earlier leaf senescence and lower yields observed for ZD958.

Practices that increase planting densities can effectively improve maize yields regionally and achieve high yields on a worldwide scale^33,34^. Maize planting densities are being increased year by year in many maize producing countries, including the United States and China^35^. However, some researchers have discovered that excessively high planting densities negatively affect the light and temperature environment within the maize canopy^14,15,20^. Improved maize yields are best achieved by instituting reasonable planting densities and improving yield per plant^11,35^. Climate conditions in different maize planting areas in China differ significantly and influence optimal maize planting densities in these regions. Maize planting densities in northwestern China are significantly higher than in other regions, which is consistent with the distribution of solar radiation in China^14,36^. In this study, maize plots were subjected to different degrees of shading that simulated different degrees of solar radiation in different maize planting regions. Our results showed that for different genotypes and different levels of solar radiation during grain filling, the planting densities that achieved the highest yields were different. In areas where solar radiation during grain filling is less than 615 MJ m^−2^ for XY335 and 630 MJ m^−2^ for ZD958, increasing planting density does not increase yields. When solar radiation during grain filling was less than 486.9 MJ m^−2^ for XY335 and 510.9 MJ m^−2^ for ZD958, yields followed the order of D1 > D2 > D3 and the best planting density was 75,000 plants ha^−1^, with average yields of 13.36 Mg ha^−1^ and 11.09 Mg ha^−1^, respectively; when solar radiation during grain filling was higher than 549.5 MJ m^−2^ and 605.8 MJ m^−2^, respectively, yields decreased in the order D3 > D2 > D1 and the appropriate planting density was 120,000 plants ha^−1^, with average yields of 20.58 Mg ha^−1^ and 19.65 Mg ha^−1^. With sufficient solar radiation, maize yields at the D3 planting density continued to increase. In contrast, yields at the D1 and D2 planting densities decreased as solar radiation increased. Several reasons could explain this phenomenon. On the one hand, when solar radiation is not a limiting factor, a high planting density can improve light interception; while at low planting densities, excessive solar radiation can result in photoinhibition and decreasing yields^37^. On the other hand, under conditions of limited solar radiation, assimilation at the D1 and D2 planting densities primarily achieves grain filling, whereas assimilation at the D3 density primarily achieves plant maintenance. Therefore, within a narrow range, low planting densities can produce higher yields than high planting densities under low solar radiation conditions^14,36^.

Previous studies have shown that different hybrids and their parents perform differently under weak-light stress. However, most weak-light resistant hybrids tend to be more tolerant of high planting densities^25,38–42^. Some researchers have found that flat-type hybrids are more sensitive to shading than compact hybrids^20,43^. The current study showed that different maize hybrids responded differently to different levels of solar radiation. Under the same solar radiation conditions, XY335 yields were higher than ZD958^27^. Yield differences between the two cultivars were mainly influenced by solar radiation and not by growth duration, as evidenced by the fact that ZD958 produced lower yields than XY335 even though ZD958 has a longer growth duration. ZD958’s yield under S-30% at low planting density, as well as under S-15% at medium planting density, was significantly lower than the CK condition. However, yield reductions for XY335 under the same conditions were not significant^27^. In the current study, maize yield decreased significantly with shading and the degree of decrease differed by cultivar. Yields of the two hybrids increased as solar radiation increased during grain filling. XY335 utilized weak light more efficiently than ZD958 and showed a greater potential to increase yield under high solar radiation conditions. The findings in this study can be useful not only to maize producers in regions with different solar radiation conditions but also to plant breeders.

In recent years, global warming caused many problems, such as drought, high temperature, and decreased solar radiation etc^17–20^^,^^44,45^. Global total solar radiation has shown a downward trend as global warming increases, and climate change has reduced maize yields. In China, for every 10% decrease in solar radiation, the average reduction in maize yields is 9.1%^46^. When solar radiation is not a constraint, grain yields can be improved by increasing planting densities. This study shows that it is possible to mitigate the effects of reduced solar radiation by selecting low-light tolerant cultivars and moderately reducing planting densities. These findings provide theoretical knowledge for achieving high-yield and high-efficiency cultivation of maize in response to future climate change conditions.

## Materials and Methods

### Experimental design

This experiment was conducted in 2016 and 2017 at Qitai Farm (Qitai, Xinjiang Uygur Autonomous Region, China) and Ningxia University Farm (Yinchuan, Ningxia Hui Autonomous Region, China). Two widely grown maize cultivars, zhengdan 958 (ZD958) and xianyu 335 (XY335), were used in this research. Only ZD958 was planted at Yinchuan in 2016. Dates for the different stages of phenological development at Qitai and Yinchuan in 2016 and 2017 are listed in Table 1. After shading, the quality of incident light in the maize canopy has been found to remain unchanged^47,48^. The geographical locations of the sites and their climatic conditions during the two maize growing seasons are listed in Table 2. Meteorological data for the 2016 and 2017 maize growing seasons were obtained from meteorological stations located near the experimental sites.

We used scaffolding and shading nets to build temporary shading sheds. Shading nets of different shade strength were designed and fabricated. The amount of solar radiation reaching a distance of 1.5 m underneath each shading net was measured with a Sunscan (SUNSCAN, Delta-T, UK) and the nets that achieved shading of 15%, 30%, and 50% of natural light were chosen for the experiments. The shading sheds were fixed in place about 1.5 m above the maize canopy in order to maintain the same microclimate conditions (except for solar radiation) observed in the unshaded portions of the field. The shading treatment period began at the silking stage and lasted until maturity because this is the key growth period that determines maize yield and this period is rainy season and decreases in solar radiation often occur during this period^47,48^. The silking stage was defined as the date when 60% of the ears showed silk emergence. Physiological maturity was defined as the date when the black layer appeared.

A split block design was used in this experiment, in which the main factor, side factor, and accessory factor were cultivar, planting density, and level of solar radiation, respectively. Maize was planted at three densities: 7.5 × 10^4^ (D1), 10.5 × 10^4^ (D2), and 12 × 10^4^ (D3) plants ha^−1^. These densities were chosen because D1 is currently recommended in many regions of China, and because D2 and D3 are known to produce higher yields^36,49^. The shading levels were 50% of natural light (S-50%), 30% (S-30%), 15% (S-15%), and no shading (CK).

Different planting densities can provide a better understanding of the interactions between solar radiation, density, and cultivars. The experimental plots were 11 × 10 m^2^ in size and each plot included 22 rows that were 9 m long. A 1 m wide walkway was set up between adjacent plots. Borders around each plot consisted of three additional rows on each of two sides of the plot, plus a 1 m long extension at the beginning and end of every row^50^. Sufficient water was applied to prevent water stress. Base fertilizers were applied at rates of 75, 150 kg ha^−1^ N as urea, 188, 225 kg ha^−1^ P_2_O_5_ (super phosphate), and 53, 75 kg ha^−1^ K_2_O (potassium sulfate) prior to sowing. An additional 300 kg ha^−1^ N was applied at both Yinchuan and Qitai during the growing season to prevent nutrient stress^50^. Weeds, diseases, and pests were controlled in the plots.

### Sampling and measurements

At the physiological maturity stage, an area of 8 m^2^ was harvested manually from the center of each plot (central four rows, 2 m long) and grain weight was measured. The grain moisture content was determined using a portable moisture meter (PM8188, Kett Electric Laboratory, Tokyo, Japan). Grain yield was determined at 14% moisture content. Aboveground dry matter at physiological maturity was measured by sampling five successive plants in the center row of each plot. Plant samples were dried at 80 °C to a constant weight. Leaf area was determined after 40 days of shading using a Portable Laser Leaf Area Analyser (CI-203; CID Bio-Science, US).

### Statistical analysis

Statistical calculations were performed using Microsoft Excel (Office 2010). Analysis of variance (ANOVA) was used to test the differences between grain yield, biomass, and leaf area index (LAI) under the different shade conditions, planting densities, cultivars, sites, and years using SPSS ver. 21.0 (SPSS Institute Inc., US). Data analysis steps were as follows: (1) the treatments and the corresponding yield data were input into SPSS data analysis view and (2) “One-Way ANOVA” analysis at a 0.05 level of probability followed by Duncan test method was chosen to compare these differences.

## References

[1] Thornton PK, van de Steeg J, Notenbaert A, Herrero M (2009). The impacts of climate change on livestock and livestock systems in developing countries: A review of what we know and what we need to know. Agr. Syst..

[2] Yang D (2017). Analysis of reason for recent slowing maize yield increase under climate change in China. Trans. Chin. Soc. Agric. Eng..

[3] Feng Z (2007). Future Food Security and Arable land guarantee for population development in China. Pop. Res..

[4] Meng Q (2011). Alternative cropping systems for sustainable water and nitrogen use in the North China Plain. Agric. Ecosyst. Environ..

[5] Zhao J, Wang R (2009). Factors promoting the steady increase of american maize production and their enlightenments for China. J. Maize Sci.

[6] Hou P (2014). Temporal and spatial variation in accumulated temperature requirements of maize. Field Crops Res..

[7] Liu Y (2013). Spatial adaptabilities of spring maize to variation of climatic conditions. Crop Sci..

[8] Wilson DR, Muchow RC, Murgatroyd CJ (1995). Model analysis of temperature and solar radiation limitations to maize potential productivity in a cool climate. Field Crops Res..

[9] Deng N (2015). Influence of temperature and solar radiation on grain yield and quality in irrigated rice system. Eur. J. Agron..

[10] Shao Z, Zhou T, Shi P, Gong D (2009). Spatial-Temoral characteristics of the influence atmospheric pollutant on surface solar radiation for Chinese key cities. Plateau. Meteor..

[11] Sher A (2017). Response of maize grown under high plant density; performance, issues and management - a critical review. Adv. Crop. Sci. Tech..

[12] Iizumi T, Ramankutty N (2015). How do weather and climate influence cropping area and intensity?. Glob. Food. Secur..

[13] Jia, S. Effects of shading on physiological characteristics of yield and quality in different genotypes maize (Zea mays L.) . *Shandong Agric Univ*, Shandong, China (2007).

[14] Xu W (2017). Adjusting maize plant density to different climatic conditions across a large longitudinal distance in China. Field Crops Res..

[15] Liu G (2017). Canopy characteristics of high-yield maize with yield potential of 22.5 Mg ha^−1^. Field Crops Res..

[16] Liu, G., *et al*. (2019). Nitrogen uptake and response to radiation distribution in the canopy of high-yield maize. *Crop Sci*. **09**, 0567, 10.2135/cropsci 0567 (2018).

[17] Li S, Wang C (2008). Analysis on change of production and factors promoting yield increase of corn in China. J. Maize. Sci..

[18] Che H (2005). Analysis of 40 years of solar radiation data from China. Geophys. Res. Lett..

[19] Ramanathan V, Feng Y (2009). Air pollution, greenhouse gases and climate change: Global and regional perspectives. Atmos. Environ..

[20] Setter TL, Flannigan BA, Melkonian J (2001). Loss of kernel set due to water deficit and shade in maize. Crop. Sci..

[21] Zhang, J. Effects of light & temperature stress on physiological characteristics of yield and quality in maize (Zea Mays L.). *Shandong Agric Univ*, Shandong, China (2005).

[22] Zhao J, Chen G (1990). Effects of shading treatment at different stages of plant development on grain production of gorn and (Zea Mays L.) observations of tip kernal abortion. Sci. Agric. Sin..

[23] Zhang J, Dong S, Wang K, Hu C, Liu P (2008). Effects of shading on dry matter accumulation and nutrient absorption of summer maize. Acta. Agron. Sin..

[24] Cui H (2013). Effects of shading on dry matter accumulation and nutrient absorption of summer maize. Chin. J. Appl. Ecol..

[25] Early EB, Mcilrath WO, Seif RD, Hageman RH (1967). Effects of shade applied at different stages of plant development on corn (Zea mays L.) Production. Crop Sci..

[26] Zhou, Z. Effect of Low Light Stress on Growth of Different Density-tolerant Varieties of Maize. *Shenyang Agric Univ*, Shenyang, China (2016).

[27] Li C, Zhao Y, Wang Q, Luan L, Li N (2005). Effects of Shading on the Senescence of Leaves and Yield of Different Genotype Maize. J. Maize Sci.

[28] Ren B (2016). Effects of shading on the photosynthetic characteristics and mesophyll cell ultrastructure of summer maize. SCI Nat-Heidelberg..

[29] Wang Y, Xiao N, Shao J, Liu Z, Li Z (2008). Effects of light intensity at full growing stage on the growth and yield of different maize varieties. J. Jilin Agric. Univ.

[30] Jia G (2017). Effect of different light intensities on root characteristics and grain yield of summer maize (Zea Mays L.). Sci. Agric. Sin..

[31] Francone C, Pagani V, Foi M, Cappelli G, Confalonieri R (2014). Comparison of leaf area index estimates by ceptometer and PocketLAI smart app in canopies with different structures. Field Crops Res..

[32] Meng QF, Cui ZL, Yang HS, Zhang FS, Chen XP (2017). Establishing High-Yielding Maize System for Sustainable Intensification in China. Adv. Agron..

[33] Assefa Y (2016). Yield responses to planting density for US modern corn hybrids. a synthesis-analysis. Crop Sci..

[34] Cui H (2013). Effects of shading on photosynthetic characteristics and xanthophyll cycle of summer maize in the field. Acta. Agron. Sin.

[35] Assefa Y (2018). Analysis of long term study indicates both agronomic optimal plant density and increase maize yield per plant contributed to yield gain. Sci Rep-UK..

[36] Ming B (2017). Changes of maize planting density in China. Sci. Agric. Sin..

[37] Wang Y, Cui Z, Zhu Y, Fan J, Zhang L (2012). The comparison of anatomic structure and photoinhibition characteristics between different regions of the C4 photosynthetic leaf in maize (Zea mays L.). Acta Phytophysiol. Sin..

[38] Fu J (2009). Effects of low-light stress on photosynthetic pigment of different maize genotypes. J. Henan Agric. Sci..

[39] Liu WD, Tollenaar M (2009). Physiological mechanisms underlying heterosis for shade tolerance in maize. Crop Sci..

[40] Moss D, Stinson H (1961). Differential response of corn hybrids to shade. Crop Sci..

[41] Stinson H, Moss D (1960). Some effects of shade upon corn hybrids tolerant and intolerant of dense planting. Agron. J..

[42] Yuan L, Li C, Wang X, Yang S (2008). Comparison of shade-tolerance among different maize (Zea mays L.). J. Maize Sci..

[43] Wang X, Liu T, Li C, Li D (2010). Effects of shading on agroncmictraits and ear development of maize cultivars (Zea mays L.) with different plant types. Acta Agric. Jiangxi..

[44] Saud, S. *et al*. Silicon application increases drought tolerance of kentucky bluegrass by improving plant water relations and morphophysiological functions. *Sci World J*. 10.1155/2014/368694 (2014) 10.1155/2014/368694 PMC4098892 25054178

[45] Saud S (2016). Silicate application increases the photosynthesis and its associated metabolic activities in Kentucky bluegrass under drought stress and post-drought recovery. Environ. Sci. Pollut. Res..

[46] Liu W, Xiong W, Wen X, Feng L (2014). Effect of climatic factors such as temperature, precipitation on maize production in China. Trans. Csae..

[47] Andrade FH, Otegui ME, Vega C (2000). Intercepted radiation at flowering and kernel number in maize. Semigroup Forum..

[48] Jia SF, Li CF, Dong ST, Zhang JW (2011). Effects of shading at different stages after anthesis on maize grain weight and quality at cytology level. Jinteg Agr..

[49] Li C (2007). Effects of shading on phomsynthetic characteristics of different genotype maize. Chin. J. Appl. Ecol..

[50] Zhang G (2017). Optimizing water use efficiency and economic return of super high yield spring maize under drip irrigation and plastic mulching in arid areas of China. Field Crops Res..

